# Fluoride Intake through Consumption of Tap Water and Bottled Water in Belgium

**DOI:** 10.3390/ijerph6051676

**Published:** 2009-05-15

**Authors:** Stefanie Vandevijvere, Benoit Horion, Michel Fondu, Marie-Josée Mozin, Michèle Ulens, Inge Huybrechts, Herman van Oyen, Alfred Noirfalise

**Affiliations:** 1 Scientific Institute of Public Health, Unit of Epidemiology, J. Wytsmanstraat 14, 1050 Brussels, Belgium; E-Mail: herman.vanoyen@iph.fgov.be; 2 Federal Public Service of Health, Food Chain Safety and Environment, Victor Hortaplein 40, 1060 Brussels, Belgium; E-Mail: benoit.horion@health.fgov.be; 3 Institute for European Studies, Université Libre de Bruxelles, Pleinlaan 2, 1050 Brussels, Belgium; E-Mail: michel.fondu@versateladsl.be; 4 Hospital for Children Queen Fabiola, Université Libre de Bruxelles, Avenue J. Crocq 15, 1020 Laeken, Belgium; E-Mail: marie-josee.mozin@huderf.be; 5 Superior Health Council, Zelfbestuurstraat 4, 1050 Brussels, Belgium; E-Mails: michele.ulens@health.fgov.be (M.U.); alfred.noirfalise@health.fgov.be (A.N.); 6 Ghent University, Department of Public Health, De Pintelaan 185, 9000 Ghent, Belgium; E-Mail: inge.huybrechts@ugent.be

**Keywords:** fluoride, exposure assessment, food consumption survey, Belgium

## Abstract

There is a tendency to align higher levels of fluoride in natural mineral water with the existing higher levels in tap water. Treatment of natural mineral waters could harm the preservation of their natural character. In this study fluoride intake through bottled and tap water consumption in the Belgian adult population was assessed, taking into account regional differences. A deterministic approach was used whereby consumption quantities of tap water and different brands of bottled water were linked with their respective fluoride concentrations. Data from the national food consumption survey (2004) were used and the Nusser methodology was applied to obtain usual intake estimates.

Mean intake of fluoride through total water consumption in Flanders was 1.4±0.7 mg/day (97.5^th^ percentile: 3.1 mg/day), while in the Walloon region it was on average 0.9±0.6 mg/day (97.5^th^ percentile: 2.4 mg/day). The probability of exceeding the UL of 7 mg per day via a normal diet was estimated to be low. Consequently, there is no need to revise the existing norms, but higher fluoride concentrations should be more clearly indicated on the labels. Reliable data about total dietary fluoride intake in children, including intake of fluoride via tooth paste and food supplements, are needed.

## 1. Introduction

Fluorides are ubiquitous in air, water and the lithosphere, where they are seventeenth in the order of frequency of occurrence (0.06–0.09% of the Earth’s crust) [1]. Fluoride in air exists in gaseous or particulate forms and arises from fluoride containing soils, industry, coal fires and volcanoes. In nonindustrial areas, concentrations range between 0.05–1.9 μg/m^3^.

Inhalation of fluoride from the air does not contribute more than 0.01 mg/day to the total intake, except in occupational settings where intake by inhalation can be several milligrams [2]. Availability of fluoride from soil depends on solubility of the fluoride compound, the acidity of the soil and the presence of water.

Fluoride in the body is mainly associated with calcified tissue (bone and teeth) due to its high affinity for calcium. Absorbed fluoride is partly retained in bone and partly excreted, predominantly via the kidneys. In infants and young children, retention in bone can be as high as 75% of the absorbed amount, whereas in adults retention is usually 50% or less [3,4]. Fluoride is also incorporated into dental enamel during tooth formation.

Fluoride is not essential for human growth and development but is beneficial in the prevention of dental caries (tooth decay) [5–8] when ingested in amounts of about 0.05 mg/kg bodyweight per day and when applied topically via dental products such as toothpaste [9–11].

Excessive intake of fluoride during enamel maturation before tooth eruption from birth to eight years of age (when enamel formation is complete) can lead to reduced mineral content of enamel and to dental fluorosis. The incidence and severity of dental fluorosis is dose-dependent [1]. The EFSA Panel considered moderate dental fluorosis, which is characterized by staining and minute pitting of teeth, to be an adverse effect. On the basis that the prevalence of moderate dental fluorosis of permanent teeth is less than 5% in populations ingesting 0.08–0.12 mg/kg bodyweight/day, the Panel considered that the upper level (UL) for fluoride is 0.1 mg fluoride/kg/day in children aged 1–8 years. This is equivalent to 1.5 and 2.5 mg fluoride per day in children aged 1–3 years and 4–8 years respectively [12].

A study with therapeutic oral administration of fluoride in amounts of 0.6 mg/kg bodyweight/day in postmenopausal women over several years increased the risk for non-vertebral bone fractures significantly [13].The EFSA Panel applied an uncertainty factor of 5 to derive an UL of 0.12 mg/kg bodyweight/day.

This is equivalent to an UL of 5 mg/day in children aged 9–14 years and 7 mg/day for adolescents and adults, aged 15 years and older, including pregnant and lactating women. The UL for fluoride applies to intake from water, beverages, foods, including fluoridated salt, dental health products and fluoride tablets for caries prevention [12].

Among the main sources of total fluoride intake in Europe are drinking waters containing more than 0.3 mg/L of fluoride [11]. Fluoride concentrations in tap water collected during 1985 from public water plants in The Netherlands were between 0.04 and 0.23 mg/L [14]. Whereas drinking water for human consumption, according to Directive 98/83/EC, may not contain more than 1.5 mg fluoride/L, natural mineral waters can have higher fluoride concentration levels. Natural mineral waters which contain more than 1 mg fluoride/L can be labeled as “contains fluoride”. According to Directive 2003/40/EC, the fluoride concentration of natural mineral waters must be less than 5 mg/L. Mineral waters exceeding 1.5 mg fluoride/L shall bear on the label the words “contains more than 1.5 mg/L fluoride; not suitable for regular consumption by infants and children under 7 years of age” and shall indicate the actual fluoride content. Twenty-four mineral waters available in Belgium were found to have fluoride concentrations below 1 mg/L in 16 cases. The highest value found was 5.5 mg/L. A case of dental fluorosis in an eight years old girl was attributed to the preparation of her infant formula with mineral water containing 1.2 mg fluoride/L [15].

Several studies show that excessive long term fluoride intake through water consumption can lead to a range of adverse effects on health such as skeletal and dental fluorosis [16–21].

Fluoride intake from food is generally low. An exception to the low fluoride concentrations found in most foods is tea, which can contain considerable amounts of fluoride (0.34–5.2 mg/L) [22,23], dependent on the type of tea and the duration of brewing. Some brands of instant teas were reported to be another significant source of fluoride intake (up to 6.5 mg/L when prepared with distilled water) [24].

Dental products which contain fluoride can, especially when inappropriately used, increase the total intake of fluoride considerably [9]. This is particularly the case in young children who swallow between 10 to 100% of their toothpaste [25–27]. In the European Communities about 90% of all toothpastes are fluoridated with a maximum level of 1,500 mg/kg.

The European legislation and the norm of the *Codex alimentarius* identified a list of chemical contaminants, fluoride included, which can be present in natural mineral water and determined upper levels for their presence. These levels often were determined using incomplete scientific data. In the near future, these norms will be revised. There is a tendency to align the upper levels of these chemical substances in natural mineral water with the existing upper levels for these substances in tap water. One of the consequences of this alignment will be that natural mineral waters will undergo the same treatment as tap water, which could harm the preservation of their natural character. Natural mineral waters contribute to a policy of sustainable development and to the protection of the natural resources. Therefore it is important in one way to preserve their natural character but in another way it is also primordial to protect the safety of the consumers using evidence-based scientific data.

The objective of this paper is to assess the intake of fluoride through consumption of bottled and tap water in the Belgian adult population, taking into account regional differences. On the basis of these results, the existing norms can be evaluated and eventual recommendations for revision of these norms can be formulated.

## 2. Design and Methods

### 2.1. Study Design

The fluoride exposure through consumption of non-alcoholic drinks was calculated using a deterministic approach where consumption quantities of tap water and different brands of bottled water were linked with their respective fluoride concentrations. Regional differences in tap water concentrations were taken into account. Fluoride concentrations of different brands of bottled water were obtained through literature, monitoring data and personal communications.

Fluoride exposure through inhalation from the air, supplements, tooth paste and salt were not taken into account because of the lack of information and because these sources of exposure are assumed to be negligible in adults in Belgium. For tea, no information was available about the duration of brewing from the food consumption survey. Fluoride exposure through food consumption was not taken into account either, but is not negligible. The French Agency for Safety of the Food Chain (AFSSA) estimates that the intake of fluoride through food consumption (water, tooth paste and supplements excluded) is about 2 mg/day for adults [28]. In the UK the mean daily fluoride intake, when including tea but excluding water, amounts to 1.2 mg/day for the adult population [29].

In this study, it is assumed that for the preparation of soft drinks and fruit juices and for the preparation of coffee, tea and broth at home, tap water was used. Tap water used for cooking or preparing meals and for brushing teeth, was not taken into account.

### 2.2. Food Consumption Data

Consumption data from the 2004 National Food Consumption Survey were used to perform the exposure assessment. Aims, design and methods used in this survey are described elsewhere [30]. The target population comprised all Belgian inhabitants of 15 years or older. The sample included 3,245 participants randomly selected from the National Register. The sampling method followed a multistage stratified procedure.

Information on dietary intake was collected by a repeated non-consecutive 24h recall in combination with a food frequency questionnaire. During the 24h dietary recalls the respondent reported the quantity of all foods and beverages consumed during the preceding day. In order to get more information on the within-person variation, two non-consecutive 24h recalls of each respondent were collected. The 24h recall was carried out using the EPIC-SOFT program [31]. This program allows obtaining very detailed information about the foods consumed up to brand level and the recipes used in a standardized way.

A total of 3,083 participants completed two 24-hour recalls of which 1,537 women and 1,546 men. Participants were categorized into four age groups: 15–18 years (n = 760), 19–59 years (n = 830), 60–74 years (n = 789) and 75 years or older (n = 704).

### 2.3. Fluoride Concentrations of Tap Water and Bottled Water

Mean and maximum concentrations of fluoride in tap water (year 2007) were obtained from the three regional distributers in Belgium (DGARNE for the Walloon region, IBGE for Brussels and VVM for Flanders). The legal norm for fluoride concentration in tap water is 1.5 mg/L (Directive 98/93/CE). Mean and maximum concentrations of fluoride in tap water differed substantially and amounted to 0.08 mg/L and 1.24 mg/L, respectively, for the Walloon region, 0.14 mg/L and 1.39 mg/L, respectively, for the Flemish region and 0.07 and 0.08, respectively, for Brussels. It was decided to use the maximum concentrations in the exposure assessment in order to provide a conservative estimate of the fluoride exposure in Belgian adults.

Fluoride concentrations of different brands of bottled water were obtained from literature [15], from monitoring data by the Belgian Federal Agency for Safety of the Food Chain (FAVV), from monitoring by the French Agency for Safety of the Food Chain (AFSSA) and through personal communications.

When taking into account consumption frequency of different brands of bottled water in Belgium, it was found that in 52.8% of the cases, the consumed brand had a fluoride concentration lower than 0.5 mg/L, in 14.6% of the cases, the fluoride concentration was between 0.5 and 1 mg/L, in 10.4% of the cases between 1 and 1.5 mg/L, in 3.7% of the cases between 1.5 and 5 mg/L and in 0.5% of the cases, the fluoride concentration was higher or equal to 5 mg/L. In 18% of the cases, the fluoride concentration of the consumed water brand could not be retrieved. It was decided to assign a fluoride concentration of 0.4 mg/L to these brands, which represents the mean concentration of the other consumed water brands, weighted by their consumption frequency. The maximum concentration of 5.5 mg/L was not assigned because this would lead to an unrealistic overestimation of the fluoride exposure in Belgium.

### 2.4. Statistical Analysis

Only respondents with two completed 24h recall interviews were included in the analyses (n = 3,083; 1,546 men and 1,537 women).

The individual intake of fluoride through consumption of non-alcoholic beverages was estimated using the following equation:

$${\text{Y}}_{\text{i}}{\;(\text{mg}/\text{day) }=\text{ C x X}}_{\text{i}}$$

where Y_i_ is the intake of fluoride by individual i from a particular type of water (in mg per interview day), C is the concentration of fluoride in that particular type of water (in mg per L), X_i_ is the consumption quantity of a certain type of water by individual *i* (in L). To estimate the total intake of fluoride per interview day, individual daily intakes of fluoride from different types of water and other beverages were added up.

The usual intake distribution for fluoride was estimated with the Nusser method [32] using the C-side software [33]. Several statistical methods are available to estimate usual intake distributions with the correct mean, variance and skewness. These statistical procedures adjust for day-to-day variability. Of all the different statistical procedures, the Nusser method [32] is highly recommended because it eliminates the intra-individual variance and additionally transforms the data to obtain approximately normally distributed data. The usual intake distribution was weighted and adjusted for the age and sex distribution of the Belgian population and adjusted for day of the week and season.

The results were stratified by region. It was not possible to calculate the results for the region of Brussels separately using C-SIDE because of the low number of positive observations for certain food groups (e.g. coffee/tea). Therefore it was decided to tabulate the results for Flanders (n = 1,923) and the Walloon region (including Brussels) (n = 1,160) separately.

## 3. Results

In Table 1 and Table 2 the mean daily consumption of non-alcoholic beverages for Flanders and the Walloon region (including Brussels) is tabulated. Total consumption of non-alcoholic beverages was higher in Flanders (p < 0.001), while total consumption of bottled water (sum of mineral and source water) was higher in the Walloon region (p < 0.05). Consumption of soft drinks (p < 0.001), coffee and tea (p < 0.001) was higher in Flanders.

Total mean intake of fluoride through consumption of non-alcoholic beverages in Flanders was 1.36±0.66 mg/day, while in the Walloon region it amounted to an average of 0.93±0.60 mg/day (p < 0.001). Fluoride intake through consumption of non-alcoholic beverages was found to be 3.06 mg/day at the 97.5^th^ percentile for Flanders and 2.44 mg/day at the 97.5^th^ percentile for the Walloon region. In both regions, the fluoride intake was higher in men than in women (p < 0.001) and individuals in the 19–59 year age group had a higher fluoride intake compared to the other age groups (p < 0.001) (Table 3 and 4).

**Figure 1:**
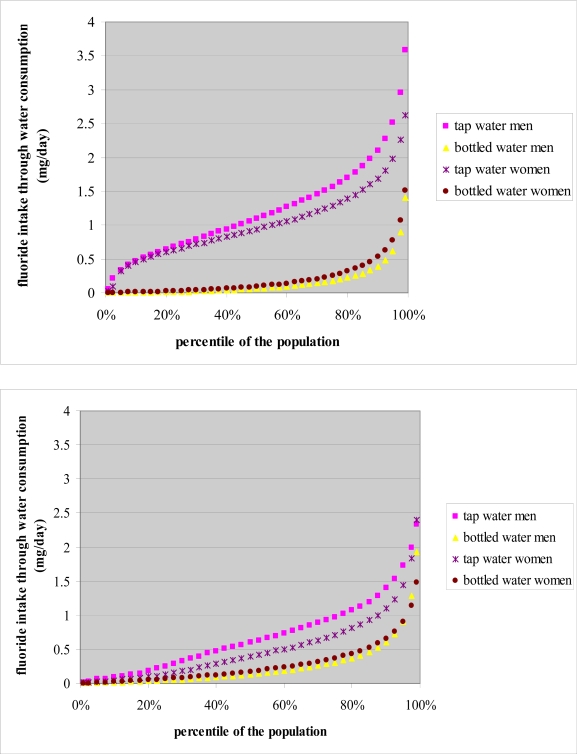
Fluoride intake (mg/day) through consumption of tap water and bottled water by sex, for Flanders (up) and the Walloon region (including Brussels) (below).

**Figure 2:**
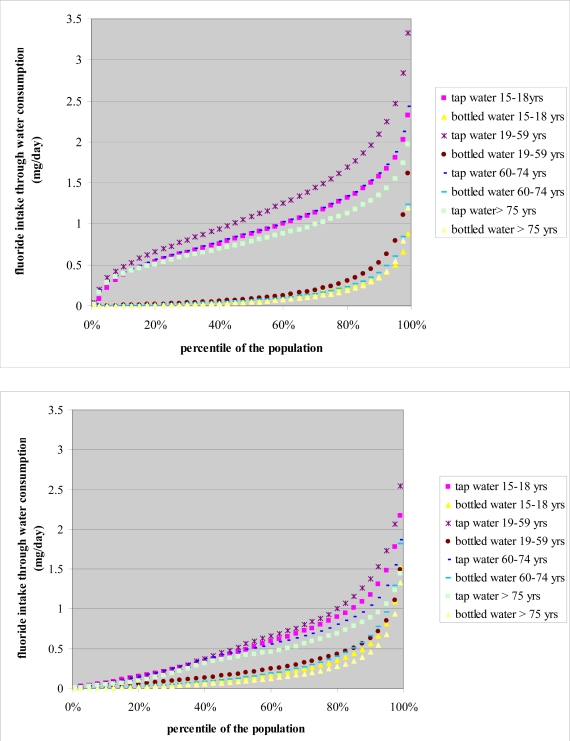
Fluoride intake (mg/day) through consumption of tap water and bottled water by age group, for Flanders (up) and the Walloon region (including Brussels) (below).

In Figures 1 and 2, it may be observed that for both regions, both sexes and all age groups the relative contribution of tap water consumption to the total fluoride intake was higher than the contribution of bottled water consumption. Fluoride exposure through consumption of tap water was higher in men than in women while fluoride exposure through consumption of bottled water was higher in women than in men in both regions. In both regions, the 19–59 year age group had the highest exposure to fluoride both through consumption of tap water and consumption of bottled water.

## 4. Discussion

In general, total consumption of bottled water was higher in the Walloon region and total consumption of tap water was higher in Flanders. People living in the Walloon region have a lower fluoride exposure compared to persons living in Flanders.

The observed fluoride intakes in both regions in the adult population were far below the upper level of intake of 7 mg/day, set by EFSA [12]. Even at the higher percentiles and taking into account an additional daily intake of fluoride through food consumption of 2 mg/day [28], the upper limit was not exceeded. Moreover, because maximum concentrations were used for the fluoride concentrations in tap water, this may have produced a considerable overestimation of the exposure.

The maximum concentrations were observed only in very specific, small areas in Belgium. On the other hand, fluoride levels in tea can be very high but were not taken into account because there was a lack of information about the duration of brewing in the food consumption survey. This may have produced an underestimation of total fluoride exposure. Also the neglect of water consumption during teeth brushing and preparation of food, and the neglect of fluoride intake through supplementation and inhalation may have posed an underestimation of fluoride intake.

On the basis of these results, we can assume that there is no potential risk for an excessive intake of fluoride in the general adult population in Belgium. In certain individual cases an excessive exposure could still occur, for example a person meeting the recommendation of consuming at least 1.5 L water a day through consumption of bottled water and being loyal to one brand containing 5 mg/L of fluoride, will exceed the upper level only through water consumption. Individuals consuming bottled water containing a fluoride concentration between 1.5 and 5 mg/L and meeting the recommendation of consuming at least 1.5 mg/L water a day, could exceed the upper level, when also fluoride intake through food consumption would be considered. Inadvertent use of highly mineralized water or unawareness of the fluoride content due to incomplete labeling, can lead to an increase of the risk for fluorosis and other health problems. Heavily mineralized water of the *Vichy* sources is known to cause fluorosis of not only the dentition [34], but also of the skeleton.

The most recent available exposure estimates to fluoride from all sources in Europe show total intakes from 0.5 to 1.2 mg/day, when no fluoridated salt or fluoride containing tooth paste are used, and no supplements are taken. In case where fluoridated salt is used and fluoridated water is drunk and used for the preparation of food and tea, the sum of fluoride intake could reach 6 mg/day, without taking into account toothpaste use [12].

A limitation of the study is that only few respondents know the source of the bottled water consumed. More respondents know the exact brand of the consumed water but although the legislation states ‘one source one brand’, this is often not respected by the industry.

Another limitation of the study is the lack of data about infants and young children. Infant formula, with the exception of soy protein based formula, has low fluoride content when the powder is prepared with low mineralized water (0.01 to 0.05 mg/L). If these formulas were prepared with water containing 0.3 mg fluoride/L and a 5-kg infant drinks 800 mL, fluoride intakes of 60 μg fluoride/kg bodyweight/day or less would result.

The use of water containing 1 mg/L of fluoride would considerably increase the fluoride intake by threefold and with a fluoride concentration between 1 and 5 mg/L the upper level could easily be exceeded.

For infants and children between the age of three and five years old in the USA total daily intakes form all sources (drinking water, beverages, infant formula, cow’s milk, food, soil, supplements and toothpaste) have been estimated. For infants, in non-fluoridated areas the average intake was estimated to be 0.08 mg/kg/day while 0.11 mg/kg/day in fluoridated areas. For young children the average intakes were 0.06 mg/kg/day and 0.06 mg/kg/day respectively [35].

In the Flemish preschool children survey from 2002 (n = 696 with three completed dietary records, 2.5 to 6 years old), it has been shown that intake of tap water is 358 mL/day while intake of bottled water was 180 mL/day [36]. The upper levels for children from 1 to 8 years old being 1.5 mg (1–3 years) or 2.5 mg (4–8 years) fluoride per day can be exceeded when bottled water with a concentration of 5 mg/L is used for consumption. When use of toothpaste and supplements would be taken into account, the upper level of 2.5 mg/day could be more easily exceeded.

Levels of fluoride through supplementation in children are estimated to be 0.25 mg/day before the age of 2, 0.50 mg/day between 2 and 4 years, 0.75 mg/day between 4 and 6 years and more than 1 mg/day older than 6 years [12]. Fluoride from toothpaste swallowed by a four-year old child was found to contribute up to one third to one half of the total daily fluoride intakes of 3.6 and 2.3 mg, respectively [37].

As part of an epidemiological study on the oral health of Flemish school children, fluoride use was studied together with risk factors (medical history, tap water fluoride concentration, use of fluoride supplements, toothpaste and brushing habits). Fluorosis was present in about 10% of the 4128 children examined. Logistic regression analyses establish tooth brushing frequency and fluoride supplement use in addition to tap water fluoride concentrations above 0.7 mg/L as significant risk factors when the presence of fluorosis on at least one tooth was used as outcome variable [38].

## 5. Conclusions

The probability of exceeding the UL of 7 mg fluoride per day via a normal diet was estimated to be very low in the Belgian adult population. However, consumption of water with high fluoride content, e.g. more than 3–4 mg/L, predisposes to exceeding the UL, when also fluoride intake through consumption of foods would be considered. A revision of the norms is not necessary but it is recommended to include fluoride concentration on the labels of bottled water. Higher fluoride concentrations should be more clearly indicated. Reliable data are needed about total dietary fluoride intake in children, especially younger children, because the upper level of fluoride intake is much lower and the intake of fluoride through consumption of tooth paste and food supplements can be considerable. The incidence and severity of dental fluorosis should be monitored as an indicator of fluoride exposure during childhood.

## Figures and Tables

**Table 1:** Mean (SE, P50, P95, P97.5, P99) usual consumption of non-alcoholic beverages in Flanders (mL/day), all days, entire adult population (Food consumption survey, 2004)

	**Mean**	**SE**	**P50**	**P95**	**P97,5**	**P99**	**N**

Non alcoholic beverages *of which*	1,446	13	1,361	1,552	2,868	3,277	1,923
*Tap water*							
Fruit and vegetable juices	63	2	32	225	281	357	668
Carbonated/ soft /isotonic drinks, diluted	219	6	145	727	902	1,143	930
Coffee, tea and herbal teas	502	9	445	1,205	1,484	1,802	1,506
Coffee	409	8	348	1,062	1,321	1,663	1,350
Tea and herbal teas	84	4	0	423	562	745	463
Tap water	73	4	0	427	603	843	429
*Bottled water*							
Mineral and source water	526	10	428	1,386	1,631	1,979	1,438
Mineral and source water, non carbonated	405	9	299	1,276	1,461	1,767	1,189
Mineral and source water, carbonated	117	5	0	537	700	921	498

SE: standard error

N: number of respondents who consumed at least one item from the particular food group during at least one of the two interview days

**Table 2:** Mean (SE, P50, P95, P97.5, P99) usual consumption of non-alcoholic beverages in the Walloon region (Brussels included), all days, entire adult population (mL/day) (Food consumption survey, 2004).

	**Mean**	**SE**	**P50**	**P95**	**P97,5**	**P99**	**N**

Non alcoholic beverages *of which*	1,370	14	1,326	2,232	2,462	2,758	1,158
*Tap water*							
Fruit and vegetable juices	60	2	33	210	263	335	432
Carbonated/ soft /isotonic drinks, diluted	190	6	138	588	703	856	504
Coffee, tea and herbal teas	363	8	322	853	1,006	1,223	916
Coffee	303	8	267	787	958	1,184	832
Tea and herbal teas	49	3	0	254	324	420	203
Tap water	132	7	0	632	826	1,085	366
*Bottled water*							
Mineral and source water	550	12	492	1,315	1,493	1,713	857
Mineral and source water, non carbonated	446	11	373	1,188	1,368	1,574	755
Mineral and source water, carbonated	99	6	0	547	727	962	202

SE: standard error

N: number of respondents who consumed at least one item from the particular food group during at least one of the two interview days

**Table 3:** Fluoride intake (mg/day) through consumption of non-alcoholic beverages in Flanders, by sex and age group (Food Consumption Survey, 2004).

	**Average**	SE*10^−2^	**P50**	**P95**	**P97,5**	**P99**	**N**	**% tap water**

Total population	1.4	1.6	1.2	2.6	3.1	3.6	1923	82
Sex								
Men	1.5	2.6	1.3	2.9	3.4	4.1	971	84
Women	1.3	1.9	1.2	2.4	2.7	3.1	952	80
Age (years)								
15–18	1.2	2.3	1.1	2.1	2.4	2.7	488	80
19–59	1.5	3.5	1.3	3.0	3.4	4.1	510	81
60–74	1.2	2.3	1.1	2.1	2.4	2.7	481	83
>75	1.0	1.9	1.0	1.8	2.1	2.3	444	83

SE; standard error

n: number of respondents

**Table 4:** Fluoride intake (mg/day) through consumption of non-alcoholic beverages in the Walloon region (Brussels included), by sex and age group (Food Consumption Survey, 2004).

	**Average**	SE*10^−2^	**P50**	**P95**	**P97,5**	**P99**	**N**	**% tap water**

Total population	0.9	1.8	0.8	2.1	2.4	2.9	0	63
Sex								
Men	1.0	2.5	0.9	2.2	2.6	3.1	575	70
Women	0.9	2.5	0.8	1.9	2.3	2.7	585	58
Age (years)								
15–18	0.9	3.6	0.8	2.0	2.3	2.8	272	62
19–59	1.0	3.4	0.9	2.2	2.6	3.1	320	64
60–74	0.9	3.4	0.8	1.9	2.3	2.8	308	60
>75	0.7	2.5	0.6	1.5	1.7	1.9	260	64

SE: standard error

n: number of respondents
